# Correlation of Genotype and Perinatal Period, Time of Diagnosis and Anthropometric Data before Commencement of Recombinant Human Growth Hormone Treatment in Polish Patients with Prader–Willi Syndrome

**DOI:** 10.3390/diagnostics11050798

**Published:** 2021-04-28

**Authors:** Agnieszka Lecka-Ambroziak, Marta Wysocka-Mincewicz, Katarzyna Doleżal-Ołtarzewska, Agata Zygmunt-Górska, Teresa Żak, Anna Noczyńska, Dorota Birkholz-Walerzak, Renata Stawerska, Maciej Hilczer, Monika Obara-Moszyńska, Barbara Rabska-Pietrzak, Elżbieta Gołębiowska, Adam Dudek, Elżbieta Petriczko, Mieczysław Szalecki, on behalf of the Polish Coordination Group for rhGH Treatment

**Affiliations:** 1 Department of Endocrinology and Diabetology, The Children’s Memorial Health Institute, 04-730 Warsaw, Poland; m.wysocka@ipczd.pl (M.W.-M.); m.szalecki@ipczd.pl (M.S.); 2 Department of Paediatric and Adolescent Endocrinology, University Children’s Hospital, 30-663 Cracow, Poland; drkate@tlen.pl (K.D.-O.); endodim@cm-uj.krakow.pl (A.Z.-G.); 3 Department of Endocrinology and Diabetology of Children and Adolescents, Wroclaw Medical University, 50-368 Wroclaw, Poland; kep@usk.wroc.pl (T.Ż.); anna.noczynska@umed.wroc.pl (A.N.); 4 Department of Paediatrics, Diabetology and Endocrinology, Medical University of Gdansk, 80-952 Gdansk, Poland; debirkhol@wp.pl; 5 Department of Endocrinology and Metabolic Diseases, Polish Mother’s Memorial Hospital—Research Institute, 93-338 Lodz, Poland; renata.stawerska@umed.lodz.pl (R.S.); maciej.hilczer@umed.lodz.pl (M.H.); 6 Department of Paediatric Endocrinology and Rheumatology, Institute of Paediatrics, Poznan University of Medical Sciences, 60-572 Poznan, Poland; m.moszynska@ump.edu.pl (M.O.-M.); b.rabska@ump.edu.pl (B.R.-P.); 7 II Clinic of Paediatrics, Endocrinology and Paediatric Diabetology, Clinical Regional Hospital No 2, 35-301 Rzeszow, Poland; sekretariat@szpital2.rzeszow.pl (E.G.); dudek.ad@wp.pl (A.D.); 8 Department of Paediatrics, Endocrinology, Diabetology, Metabolic Disorders and Cardiology of Developmental Age, Pomeranian Medical University, 71-242 Szczecin, Poland; elzbieta.petriczko@pum.edu.pl; 9 Collegium Medicum, Jan Kochanowski University, 25-317 Kielce, Poland

**Keywords:** Prader–Willi syndrome, imprinting disorder, recombinant human growth hormone, insulin-like growth factor 1

## Abstract

Genotype–phenotype correlation in patients with Prader–Willi syndrome (PWS) has still not been fully described. We retrospectively analysed data of 147 patients and compared groups according to genetic diagnosis: paternal deletion of chromosome 15q11-q13 (DEL 15, *n* = 81), maternal uniparental disomy (UPD 15, *n* = 10), excluded DEL 15 (UPD 15 or imprinting centre defect, UPD/ID, *n* = 30). Group DEL 15 had an earlier genetic diagnosis and recombinant human growth hormone (rhGH) start (*p* = 0.00), with a higher insulin-like growth factor 1 (IGF1) level compared to group UPD/ID (*p* = 0.04). Among perinatal characteristics, there was only a tendency towards lower birth weight SDS in group UPD 15 (*p* = 0.06). We also compared data at rhGH start in relation to genetic diagnosis age—group 1: age ≤9 months, group 2: >9 months ≤ 2 years, group 3: > 2 years. Group 1 had the earliest rhGH start (*p* = 0.00), with lower body mass index (BMI) SDS (*p* = 0.00) and a tendency towards a higher IGF1 level compared to group 3 (*p* = 0.05). Genetic background in children with PWS is related to time of diagnosis and rhGH start, with a difference in IGF1 level before the therapy, but it seems to have little impact on perinatal data. Early genetic diagnosis leads to early rhGH treatment with favourable lower BMI SDS.

## 1. Introduction

Prader–Willi syndrome (PWS) is a rare disease with an estimated prevalence of 1 in 15,000 to 1 in 30,000. It had been previously recognised on the basis of clinical diagnostic criteria, but nowadays it should be confirmed by molecular genetic testing. PWS is a first recognised human genetic imprinting disorder and results from a lack of paternally inherited genes on chromosome 15q11-q13, which can be caused by paternal deletion (DEL 15, 65–75%), maternal uniparental disomy (UPD 15, 20–30%) or an imprinting centre defect (ID, estimated for 1–3%).

Among the imprinted genes are: *SNURF-SNRPN* (*SNRPN* upstream reading frame- small nuclear ribonucleoprotein polypeptide N), *MKRN3* (makorin RING-finger protein 3), *MAGEL2* (MAGE Family Member L2), *NDN* (Necdin protein), *C15orf2* (chromosome 15 open reading frame 2) and more than 70 C/D box snoRNA genes (*SNORDs*). The exact contribution of these genes to PWS is still not fully understood [1,2,3,4].

The main clinical features of PWS are mostly age-dependent and consist, in the first months of life, of marked hypotonia, global psychomotor delay, and moderate to severe difficulties in feeding with failure to thrive. This period is usually followed by a lack of satiety and obesity developing from early childhood that lead, if untreated, to morbid obesity. Other typical features in patients with PWS are hypogonadotropic hypogonadism, with almost universal cryptorchidism in male newborns, short stature, and cognitive and behaviour dysfunction [4,5,6,7,8,9]. However, the latest studies show a more complex phenotype in patients with PWS than had been established before. We are now more aware of heterogeneity of gonadal dysfunction, both hypogonadotropic and primary gonadal [10,11,12,13]. There are also studies confirming frequent premature adrenarche which does not influence the central puberty course or recombinant human growth hormone (rhGH) effectiveness [14]. The recent studies showed very importantly that we can also distinguish more age-specific nutritional phases in most patients with PWS [15,16,17,18,19,20]. According to Miller et al., in childhood we can describe the following phases (median age): 1a, until 9 months of life, characterised by feeding difficulties and decreased appetite; 1b, until 25 months, with improved feeding and appetite; 2a, until 4.5 years, when weight increases without increased appetite; 2b, until 8 years, with increased appetite and calorie intake. In a further phase, 3, that lasts until adulthood, patients are hyperphagic and show insatiable appetite [19]. The rhGH treatment together with multidisciplinary care has been a well-established approach to patients with PWS and it seems to positively influence not only the natural history of body composition and psychomotor development but also the above-described natural phases of PWS nutritional phenotype [21,22,23,24,25,26,27]. 

Although PWS is a rare disease, it can serve as a model of hypothalamic function disruption and, therefore, understanding PWS can help both scientists and clinicians better understand and maintain other diseases with a similar background, such as hypothalamic tumours before and after neurosurgery interventions. Moreover, we still lack full recognition of the influence of specific missing genes on hypothalamus dysfunction and its impact on PWS phenotype [2,3,4,28]. Studies regarding the relationship between the genotype and newborn phenotype have been conducted, but the results are not explicit [29,30,31,32,33]. There is also little data for anthropometric characteristics before rhGH treatment in relation to the molecular type of diagnosis. The latest research does not give concordant results regarding overall anthropometric differences between the genetic subtypes [34,35,36,37]. In the study by Butler et al. in 2019, regarding both paediatric and adult patients, with only part of them treated with rhGH, there was no difference in body mass index (BMI) identified, based on the genetic subtype [34]. In the recently published paper by Shepherd et al. in 2020, describing paediatric population, detailed information regarding rhGH treatment was not reported and no difference in height was found for males in both PWS subtypes (DEL 15 vs. non-DEL 15), with decreased height in females with non-DEL 15 for older ages. Weight and BMI were higher in the DEL 15 group, which suggests that these patients are more prone to obesity [35]. These results correspond with the findings by Mahmoud and Leonenko et al. in a multicentre study of a large cohort of 355 patients, published this year [36]. However, the authors of the above studies did not analyse the characteristics of patients with PWS with a different molecular diagnosis at the start of rhGH therapy. 

We conducted our research to try to answer the question of whether the type of molecular diagnosis significantly influences the perinatal characteristics of newborns with PWS, the time of genetic diagnosis and the age of rhGH commencement as well as anthropometric parameters and insulin-like growth factor 1 (IGF1) level before the therapy. Furthermore, we investigated the patients’ anthropometric and IGF1 values at the time of rhGH start in relation to the age of genetic diagnosis.

## 2. Material and Methods

We retrospectively analysed data (medical records and questionnaires filled in by clinicians) regarding time and type of molecular diagnosis, perinatal characteristics, and anthropometric and biochemical data before commencement of rhGH treatment in 147 patients, 69 girls (46.94%) and 78 boys (53.06%), from 12 paediatric endocrine centres in Poland, in the years 2002–2016. The rhGH treatment was accepted by the Polish Coordination Group for rhGH Treatment. 

We have grouped the patients according to the subtype of genetic diagnosis: group DEL 15, *n* = 81 (55.1%), group UPD 15, *n* = 10 (6.80%). In 20 patients, the DEL 15 has been excluded and the diagnosis can be either UPD 15 or ID, and in 7 of them (4.76% of the whole cohort) DEL15 was excluded but further molecular studies have not been documented. Therefore, we created a third group of patients, with excluded DEL 15 diagnosis, group UPD/ID, *n* = 30 (20.41%). In 36 patients (24.49%), the abnormality in methylation pattern of *SNRPN* was confirmed, but the exact type of genetic diagnosis has been pending—in 1 patient the genetic report was not included in the documentation. As the exact molecular diagnosis in this group of patients had not been established, the group can be very heterogeneous and the patients may present with all possible molecular types of PWS diagnosis. Therefore, they were not analysed separately.

Subsequently, we analysed the data regarding the rhGH start according to the age of genetic diagnosis. We were directed by the age-depended nutritional phases, as described above. We divided our cohort into 3 groups: group 1: age ≤9 months, group 2: >9 months ≤ 2 years, group 3: > 2 years of life. 

The height and BMI were assessed according to the Polish growth and BMI standards charts, and the BMI SDS was calculated using the LMS method (method to obtain SD, LMS parameters: Lambda for the skew, Mu for the median, and Sigma for the generalized coefficient of variation). IGF1 was evaluated with a radioimmunoassay technique. 

The study was approved by the CMHI Bioethics Committee, 7/KBE/2019, 20 March 2019. 

## 3. Data Analysis

Statistical analyses were performed using TIBCO Software Inc. (2017) Statistica version 13 StatSoft Company. Results are expressed as mean values and standard deviation scores (±SDS) and additionally as median (minimal-maximal value) for the age of diagnosis and therapy start. Data were checked for normality of distribution using the Shapiro–Wilk test, and data with skewness were log or square transformed to normal distribution if possible. Differences between the groups were tested by unpaired t-Student test or Mann–Whitney U test, as appropriate. Correlations between the assessed parameters were evaluated with Pearson correlation and Spearman rank correlation, dependent on distribution. Differences between the dependent variables were analysed by t-Student test for dependent variables or Wilcoxon test as appropriate. A *p* level <0.05 was recognized as statistically significant. 

## 4. Results

The details regarding the perinatal period are presented in Table 1. 

The mean values of week of gestation, Apgar score (AS) at the 1st and 10th minute of life and length SDS at gestation were within the normal range in the whole cohort (*n* = 147) and in the groups DEL 15, UPD 15 and UPD/ID. The mean birth weight was above −2 SDS, apart from the group UPD 15 (−2.69 ± 0.92). This difference was marked but did not reach statistical significance (*p* = 0.06). Below, we present the more detailed neonatal characteristics within the groups.

**Group DEL 15**—*n* = 81, 43 girls, 38 boys; 18 patients (22.22%) were born prematurely (31–37 weeks of pregnancy), most of the patients by caesarean section (49 patients, 60.49%). Thirty-six patients (44.44%) were born small for gestational age (SGA), according to the birth weight SDS, four patients according to both the birth weight and length. Thirty-two newborns (39.51%) had a lower AS of 1–7 points in the first minute of life. Six patients (28.40%) presented with serious complications within the neonatal period (mainly intrauterine infection, breathing difficulties and intraventricular haemorrhage (IVH), less often hypoglycaemia, seizures, gastrointestinal bleeding, thrombocytopenia, congenital heart defect). Cryptorchidism was present in 30 out of 38 boys (79%), and orchidopexy was performed in 23 boys, aged 2.95 ± 2.73 years. 

**Group UPD**—*n* = 10, one girl, nine boys; two patients (20%) were born prematurely (35–36 weeks of pregnancy), most of the patients by caesarean section and as SGA (eight patients, 80%) with birth weight <2 SDS, one patient with both birth weight and length <2 SDS, and five (50%) with a lower AS of 5–7 points in the first minute of life. Three patients (30%) presented with serious neonatal complications (serious breathing difficulties, IVH, intrauterine infection). Cryptorchidism was present in all boys, and eight underwent orchidopexy at the age 7.15 ± 5.49 years.

**Group UPD/ID**—*n*= 30, 11 girls, 19 boys; eight patients (26.67%) were born prematurely (30–37 weeks of pregnancy), most of the patients by caesarean section (19 patients, 63.33%) and as SGA (16 patients, 53.33%) with birth weight <−2 SDS, two patients with both birth weight and length <−2 SDS, one patient with only birth length <−2 SDS, and half of the group with a lower AS of 1–7 points in the first minute of life. Six patients (20%) presented with serious neonatal complications (serious breathing difficulties, IVH, intrauterine infection, hepatitis). Cryptorchidism was present in almost all boys (18 boys, 94.74%), and 13 of them underwent orchidopexy at the age 5.82 ± 4.83 years.

There was a statistical significant difference in the age of genetic diagnosis between the group DEL 15 and group UPD 15 (*p* = 0.003), as well as group UPD/ID (*p* = 0.00) (Table 2).

Children with DEL 15 were diagnosed much earlier, in the mean age within the 2nd year of life, whereas the groups UPD 15 and UPD/ID in the 4th year of life. The difference was even more evident when analysing the median values, with the age of diagnosis in the group DEL 15 of 5 months of age. 

There was also a later start of rhGH treatment in the groups UPD 15 and UPD/ID compared to the group DEL 15 (*p* = 0.003, *p* = 0.00). The mean height SDS was below −2 SDS in the groups UPD 15 and UPD/ID, and −1.95 SDS in group DEL 15, with the mean BMI SDS within the normal range in the whole population of patients at the beginning of the therapy. We did not confirm any statistical difference in the height and BMI SDS at the start of the treatment within the groups. However, there was a tendency towards the higher BMI SDS in the groups UPD 15 and UPD/ID (*p* = 0.14, *p* = 0.15). We found a positive correlation between the age of genetic diagnosis and BMI SDS in the group DEL 15, as well as in the group UPD/ID (*r* = 0.27, *p* = 0.014 and *r* = 0.38, *p* = 0.044, respectively), but no correlation was found with the height SDS.

Interestingly, comparison of the results of birth length and height before the start of the treatment expressed in SDS showed a higher SDS for birth length in all the groups (*p* = 0.00). 

The mean IGF1 level before rhGH therapy was close to −1 SDS in the patients from all the groups (*n* = 142), with a significantly lower IGF1 SDS value in the group UPD/ID vs. DEL 15 (*p* = 0.04). When we correlated the mean birth anthropometric parameter SDS with IGF1 SDS, we did not find the statistically significant correlations in the groups DEL 15 and UPD/ID. The details regarding the data before the rhGH start are presented in Table 2.

Furthermore, we analysed the patients’ data according to the age of genetic diagnosis: group 1: age ≤9 months, group 2: >9 months ≤2 years, group 3: >2 years of life (Table 3). 

Group 1—*n* = 82, 39 girls, 43 boys; DEL 15 was diagnosed in 51 (62.2%), UPD 15 in 2 (2.44%), UPD/ID in 8 (9.76%), abnormality in methylation pattern of *SNRPN* in 23 (28.05%) patients. Group 2—*n* = 25, 13 girls, 12 boys; DEL 15 was diagnosed in 12 (48%), UPD 15 in 2 (8%), UPD/ID in 6 (24%), abnormality in methylation pattern of *SNRPN* in 7 (28%) patients. Group 3—*n* = 40, 17 girls, 23 boys; DEL 15 was diagnosed in 18 (45%), UPD 15 in 6 (15%), UPD/ID in 16 (40%), abnormality in methylation pattern of *SNRPN* in 6 (15%) patients.

The patients with early genetic diagnosis started rhGH treatment significantly earlier than children diagnosed >9 months of age, with a lower BMI SDS than in group 3 but similar height SDS to groups 2 and 3. BMI SDS was correlated with the age of diagnosis in the oldest group (*r* = 0.41, *p* = 0.01). The IGF 1 SDS was higher in the first group compared to group 3, but did not reach statistical significance (*p* = 0.052).

As we cover 15 years in our analysis, and in those years a number of advances in diagnosis and treatment occurred, we looked in more detail at the data of patients diagnosed in the years 2002–2009: *n* = 94 (63.95%), F/M (%) 38/56 (40.43/59.57), DEL15 *n* = 51 (54.26%), UPD 15 *n* = 9 (9.58%), UPD/ID *n* = 21 (22.34%), abnormality in methylation pattern of *SNRPN n* = 22 (23.40%); and in the years 2010–2016: *n* = 53 (36.05%), F/M (%) 31/22 (58.49/41.51), DEL15 *n* = 30 (56.60%), UPD 15 *n* = 1 (1.89%), UPD/ID *n* = 9 (16.98%), abnormality in methylation pattern of *SNRPN n* = 14 (26.42%). As expected, we found a significant difference in the age of diagnosis and the age of rhGH commencement between the groups, with the older age in the patients diagnosed in the years 2002–2009 vs. 2010–2016: 1.85 ± 2.08 years, med 1.01 (0.02–7.31) vs. 1.35 ± 2.84 years, med 0.26 (0.04–12.49), *p* = 0.00 and 5.67 ± 3.68 years, med 4.53 (0.93–17.43) vs. 2.57 ± 2.97 years, med 1.63 (0.58–14.55), *p* = 0.00, respectively. Analysis of the perinatal data showed only a tendency toward higher AS in the first minute of life in the patients diagnosed in the years 2010–2016: 7.80 ± 2.06 vs. 7.11 ± 2.32, *p* = 0.08.

Among the anthropometric characteristics before rhGH start, the patients diagnosed in the years 2002–2009 presented with higher BMI SDS: 0.71 ± 1.26 vs. −0.12 ± 1.85, *p* = 0.002. 

Interestingly, height SDS as well as IGF1 SDS and rhGH dose were comparable between those groups of children.

## 5. Discussion

Compared to the recent literature, our findings show more frequent SGA characteristics among newborns with PWS. In our cohort of patients, both with DEL 15 and with UPD/ID, more than half of the patients were born by caesarean section, and half of the groups presented as SGA, mainly according to the birth weight, and with lower Apgar score. Close to 25% were born prematurely and with serious complications within the neonatal period. Among the perinatal anthropometric results, there was only a tendency towards the lower birth weight SDS in the group UPD 15. Singh et al., in their study in 2018, analysed data of 355 patients with PWS from the Rare Diseases Clinical Research Network (RDCRN) PWS registry and found that 54% were born by caesarean section, 26% were born prematurely and 34% were born with a low birth weight, and also no significant differences in the genetic subtypes were noted [29]. Bar et al., in a 2017 analysis of 61 newborns with PWS, reported 67% born by caesarean section, 20% prematurely and 30% newborns small for gestational age, and the data regarding the details within the genetic subtypes were not reported [30]. Salvatoni et al. in 2019 in a large cohort of 252 male and 244 female newborns with PWS confirmed only decreased birth length in females with DEL 15 vs. those with UPD 15 [31]. In a paper published in 2019, among 102 Chinese children with PWS, the authors observed a higher frequency of premature newborns in group UPD 15, the characteristic that was comparable in our groups of patients with DEL 15 and UPD 15 (22 and 20%). There was a difference in the frequency of cryptorchidism, with 57.3% in the group DEL 15 and 74.1% in the group UPD 15 [32]. In our cohort, cryptorchidism was present in 79% of boys in the group DEL 15, while it was almost a universal feature in the groups UPD and UPD/ID.

We presented new data regarding the age of diagnosis and the age of rhGH start in different PWS genetic subtypes. The patients in the group DEL 15 were diagnosed and started the treatment earlier in comparison to the groups UPD and UPD/ID. These data may suggest that the clinical manifestation of PWS in the case of patients with DEL 15 is more evident, even in view of the tendency to the lower mean weight SDS in the group UPD 15. However, we do not have the precise data regarding the incidence and severity of hypotonia or feeding difficulties in the neonatal period as well as dysmorphic features in all the patients. Therefore, we cannot formulate a definitive explanation for the difference in the time of diagnosis between children with different molecular diagnosis. Interestingly, the mean height and BMI SDS were closer to the normal mean values in the group DEL 15 at the start of the rhGH treatment, although these differences did not reach statistical significance. It may be explained by the earlier rhGH start, following the earlier diagnosis. It may be also speculated that the clinical, mainly dysmorphic features of PWS, other than short stature or higher BMI, are more relevant in the children with DEL 15. The DEL 15 phenotype has been described as a classic PWS phenotype that may therefore lead to the earlier diagnosis and start of the therapy [28,36]. The evident difference between the birth length SDS and height SDS before the start of the treatment in our research, independent of the genetic subtype, may indicate that there is a significant decrease in postnatal growth in children with PWS, regardless the specific molecular diagnosis. The mean IGF1 SDS values at the rhGH start were within the normal range for age and sex in all the genetic subtypes. There was a significantly lower IGF1 SDS in the group UPD/ID vs. DEL 15 that may again correspond to the later rhGH start in this group of patients. However, the height SDS did not differ between the groups. 

Our research includes a new analysis of the time of diagnosis and start of the treatment within the different nutritional phases. The early genetic diagnosis (≤9 months of age) in the period of the phenotype of hypotonia and feeding difficulties with possible failure to thrive leads to the significantly earlier rhGH start. However, the mean beginning of the treatment was still in the 3rd year of life (median age close to the end of the 2nd year), when the weight tends to increase but still without increased appetite. Children diagnosed >9 months ≤2 years started the therapy in the 5th year of life (median 4th year), close to the period of increased appetite. The patients that were diagnosed at >4.5 years started the treatment even later, in the 9th year of life, when the individuals with PWS experience hyperphagia and insatiable appetite [19]. Moreover, early genetic diagnosis leads to the possible favourable lower BMI SDS and the tendency towards a higher IGF1 level at the rhGH treatment start. It may be probably explained by the younger age when the nutritional behaviour is more appropriate for healthy children and hypothetical growth hormone deficiency is less expressed. Interestingly, the height SDS still did not show any differences at the rhGH start. In the view of the positive effects of rhGH therapy in modifying not only the anthropometric characteristics but hypothetically also the satiety and appetite behaviour, it seems to be a crucial step to diagnose PWS in the first months of life and therefore start the treatment early [38,39]. Commencement of the rhGH therapy, which has a potential beneficial influence on metabolic state, as well as on psychomotor development, before the nutritional phase of increased appetite may modify the subsequent nutritional phenotype of patients with PWS. Moreover, early diagnosis has a potential influence on better multidisciplinary care with healthier nutritional habits, even before the rhGH therapy. However, analysing our cohort of patients, we have to take into consideration that some of them were born in the years before rhGH therapy was available in Poland cost-free for children with PWS (2006). Only a few of them were treated before with families’ own resources. This may explain the discrepancy between the early diagnosis and relatively late start of the treatment in some of the patients. Although most of the patients were diagnosed in the years 2002–2009, they were significantly older at the time of genetic diagnosis as well as at the start of rhGH treatment, with a higher BMI SDS.

Finally, our research confirmed the need for specific molecular diagnosis in all patients with PWS, which is important not only for further genetic counselling but also for a better understanding of the possible future phenotype. In the analysed cohort, almost 25% of patients had the abnormality in the methylation pattern of *SNRPN*, but the exact type of genetic diagnosis has not yet been made and almost 5% of children had DEL15 excluded, but further molecular studies have not been documented. It may be partially explained by the early years of the genetic diagnostic process when not all of the molecular methods were widely available. However, the frequency of non-specific molecular diagnosis was close to 25% in the patients diagnosed in the years 2002–2009 as well as in the years 2010–2016. In a population-based observation of 160 Australian children with PWS in the years 1951–2012, published in 2015, there was a significant part of missing the exact molecular diagnosis: 58% in the years 1973–1981, with no UPD 15 diagnosis, and 17% in the years 2003–2012, with 45% of UPD 15. Similarly to our results, a quarter of the cohort was born prematurely and half of the analysed group as SGA [40]. In a lately published study showing outcomes of rhGH treatment in patients with PWS, there are also data with a high percentage of non-defined molecular diagnosis. In a large cohort of 522 prepubertal children and 173 adolescents with PWS treated with rhGH in the years 1987–2012 (Pfizer International Growth Database, KIGS), reported by Bakker et al. in 2017, the diagnosis of PWS was confirmed by genetic studies in 79% in both groups of patients, and in 14% and 20% of those, respectively, the exact genetic aberration was unknown. It seems that the remaining 21% of patients were still diagnosed on the basis of clinical criteria. The rhGH treatment was initiated at the mean age of 4.4 ± 2.9 and 8.2 ± 2.7 years, respectively [41]. Another large group of children treated with rhGH was analysed by Sävendahl et al. in 2019 and Angulo et al. in 2020 with the data from The American Norditropin Studies (ANSWER, years 2002–2016) and NordiNet International Outcome Study (NordiNet IOS, years 2006–2016, Europe) [42,43]. There were 234 and 132 paediatric patients with PWS, with the detailed analysis of 78 and 67 patients, respectively, but without reports regarding the genetic type and age of diagnosis. The mean age at baseline was 4.67 ± 5.00 and 4.91 ± 4.88 years [42]. There were no data on the anthropometric values at baseline in relation to the exact molecular diagnosis presented in the above studies regarding rhGH treatment. However, we can see that the later therapy start was more common in the previous years, similarly to our results. We acknowledge the limitations of our research. As this is a retrospective multicentre study, including the data from 15 years of patients’ observation, not all the information may be fully precise. Questionnaires were filled in by clinicians from 12 endocrine centres; therefore, no strict standardization across all providers regarding documentation was present. 

## 6. Conclusions

In our study, we presented new data regarding the influence of the genetic background in children with PWS on the time of diagnosis and rhGH start, with a significantly earlier genetic diagnosis and commencement of the therapy in children with DEL 15. This molecular type of diagnosis is also related to the possible favourable higher IGF1 level and the tendency towards lower BMI SDS before rhGH treatment. The type of genetic diagnosis seems to have little impact on perinatal data, with only the tendency towards lower birth weight SDS in the group UPD 15. 

Additionally, we presented a new analysis of the time of diagnosis and start of the therapy in regard to the different nutritional phases in patients with PWS. Diagnosis in the nutritional phase 1a, before the 9th month of age, leads to earlier rhGH treatment, with the start early in the phase 2a when increased appetite is not yet observed and again with favourable lower BMI SDS and the tendency towards a higher IGF1 level. 

In conclusion, we confirmed the importance of the early exact molecular type of diagnosis in patients with PWS and found differences in the circumstances of rhGH commencement between PWS genetic subtypes. 

## Figures and Tables

**Table 1:** Clinical characteristics at birth (the mean value ± standard deviation score, SDS).

PWS Group	All Patients	DEL 15	UPD 15	UPD/ID
Number of patients (%)	N = 147	N = 81 (55.10)	N = 10 (6.80)	N = 30 (20.41)
F/M (%)	69/78 (46.94/53.06)	43/38 (53.09/46.91)	1/9 (10/90)	11/19 (36.67/63.33)
Weeks of gestation	38.68 ± 2.65	38.76 ± 2.31	39.33 ± 2.29	38.50 ± 3.26
Apgar score 1st minute	7.36 ± 2.25	7.75 ± 1.94	7.70 ± 1.64	7.41 ± 1.82
Apgar score 10th minute	8.41 ± 1.20	8.41 ± 1.24	ND	8.27 ± 0.90
Weight [g]	2692.91 ± 534.95	2720.40 ± 527.70	2547.00 ± 471.03	2646 ± 558.21
Weight SDS	−1.88 ± 1.29	−1.85 ± 1.26	−2.69 ± 0.92 (*p* = 0.06) *	−1.91 ± 1.30
Length [cm]	52.13 ± 4.42	52.46 ± 4.16	51.20 ± 4.26	51.11 ± 5.83
Length SDS	1.20 ± 1.90	1.28 ± 1.99	0.27 ± 1.61	0.84 ± 2.15

PWS—Prader–Willi syndrome, DEL 15—deletion of chromosome 15q11-13, UPD 15—uniparental disomy, ID—imprinting defect, F/M—female/male, ND—not done. * group UPD 15 vs. DEL 15.

**Table 2:** The age of PWS genetic diagnosis and the data at the start of rhGH treatment (the mean value ± standard deviation score, SDS; median (minimal-maximal value) for the age of diagnosis and rhGH start).

PWS Group	All Patients	DEL 15	UPD 15	UPD/ID
Number of patients (%)	N = 147	N = 81 (55.10)	N = 10 (6.80)	N = 30 (20.41)
F/M (%)	69/78 (46.94/53.06)	43/38 (53.09/46.91)	1/9 (10/90)	11/19 (36.67/63.33)
Age of diagnosis [years]	1.67 ± 2.39 0.53 (0.02–12.49)	1.38 ± 2.33 0.41 (0.02–12.49)	3.84 ± 2.86 4.1 (0.13–7.31) (*p* = 0.003) *	3.13 ± 2.72 2.55 (0.09–8.84) (*p* = 0.00) *
Age of rhGH start [years]	4.55 ± 3.74 3.03 (0.58–17.43)	4.24 ± 3.81 2.64 (0.58–16.75)	7.30 ± 3.03 8.31 (3.29–10.62) (*p* = 0.003) *	6.42 ± 3.74 5.62 (0.85–17.43) (*p* = 0.00)*
rhGH dose (IU/kg/week; mg/kg/day)	0.58 ± 0.16; 0.028 ± 0.008	0.57 ± 0.14; 0.027 ± 0.007	0.61 ± 0.13; 0.029 ± 0.006	0.60 ± 0.16; 0.029 ± 0.008
Height [cm]	96.62 ± 23.31	95.23 ± 24.18	111.78 ± 15.65	107.85 ± 20.34
Height SDS	−2.11 ± 1.50	−1.95 ± 1.53	−2.45 ± 1.07	−2.18 ± 1.57
BMI	18.05 ± 3.99	17.79 ± 3.78	20.55 ± 4.24	19.31 ± 3.40
BMI SDS	0.41 ± 1.55	0.39 ± 1.61	1.16 ± 0.91 (*p* = 0.14) *	0.86 ± 1.29 (*p* = 0.15) *
IGF1 [ng/mL]	70.31 ± 55.06	75.80 ± 64.56	84.11 ± 47.63	71.93 ± 38.79
IGF1 SDS	−0.89 ± 0.43	−0.83 ± 0.46	−1.03 ± 0.55	−1.03 ± 0.43 (*p* = 0.04) **

PWS—Prader–Willi syndrome, DEL 15—deletion of chromosome 15q11-13, UPD 15—uniparental disomy, ID—imprinting defect, F/M—female/male, rhGH—recombinant human growth hormone, BMI—body mass index, IGF1—insulin-like growth factor 1. * group UPD 15 vs. DEL 15, UPD/ID vs. DEL 15, ** group UPD/ID vs. DEL 15.

**Table 3:** The age of PWS genetic diagnosis and the data at the start of rhGH treatment depending on the age of PWS molecular diagnosis (the mean value ± standard deviation score, SDS; median (minimal-maximal value) for the age of diagnosis and rhGH start).

PWS Group	Group 1	Group 2	Group 3
Number of patients (%)	*n* = 82 (55.8)	*n* = 25 (17)	*n* = 40 (27.2)
F/M (%)	39/43 (47.56/52.44)	13/12 (52/48)	17/23 (42.50/57.50)
Age of diagnosis [years]	0.25 ± 0.18 0.21 (0.02–0.71)	1.35 ± 0.36 1.22 (0.79–1.93) (*p* = 0.00) *	5.00 ± 2.56 4.33 (2.06–12.49) (*p* = 0.00) *
Age of rhGH start [years]	2.60 ± 2.28 1.97 (0.58–13.09)	4.86 ± 3.39 3.53 (1.59-12.56) (*p* = 0.00) *	8.44 ± 3.43 8.28 (2.91–17.43) (*p* = 0.00) *
rhGH dose (IU/kg/week; mg/kg/day)	0.57 ± 0.14; 0.027 ± 0.007	0.58 ± 0.15; 0.027 ± 0.007	0.60 ± 0.21; 0.029 ± 0.01
Height [cm]	84.57 ± 16.78	99.88 ± 21.04	119.81 ± 18.33
Height SDS	−2.13 ± 1.52	−2.02 ± 1.61	−2.10 ± 1.44
BMI	16.21 ± 2.60	18.79 ± 3.44	21.52 ± 4.35
BMI SDS	−0.21 ± 1.62	1.14 ± 1.26 (*p* = 0.00) **	1.24 ± 0.79 (*p* = 0.00) **
IGF1 [ng/mL]	52.46 ± 36.44	79.72 ± 64.76	101.87 ± 66.44
IGF1 SDS	−0.84 ± 0.27	−0.87 ± 0.43	−1.01 ± 0.64 (*p* = 0.052) ***

PWS—Prader–Willi syndrome, F/M—female/male, rhGH—recombinant human growth hormone, BMI—body mass index, IGF1—insulin-like growth factor 1, group 1: age ≤9 months, group 2: >9 months ≤2 years, group 3: >2 years of life, * group 2 vs. 1, group 3 vs. 1, group 3 vs. 2, ** group 2 vs. 1, group 3 vs. 1, *** group 3 vs. 1.

## Data Availability

The data presented in this study are available on request from the corresponding author. The data are not publicly available due to ethical restrictions.
